# Supplementary material for: Are carbon emissions trading and green financial instruments synergistic? -Comprehensive quantitative research based on content analysis
Source: PLoS One. 2024 Mar 7;19(3):e0298601. doi: 10.1371/journal.pone.0298601 (PMC10919601; doi:10.1371/journal.pone.0298601)
Supplement: S1 Data — (ZIP) [file pone.0298601.s001.zip › Data/地级市Z_F工作报告.zip!/╣π╢1⁄2/╜╥╤⌠╩╨/└┤╫╘╣1⁄2╓┌║┼í╛┬φ┐╦╩2╛▌═°í┐_2013.docx]

各位代表：

现在，我代表市人民政府向大会作报告，请予审议，并请政协各位委员和其他列席人员提出意见。

2012年工作回顾

过去一年，是新一届政府攻坚克难、经受考验的一年。一年来，在市委的正确领导和市人大及其常委会、市政协的监督支持下，我们紧紧围绕“打造粤东发展极，建设幸福新揭阳”核心任务，牢牢把握“强化效率、彰显公平、惠民执政、绿色发展”施政理念，在调结构、促转型、惠民生上迈出了新步伐，实现了良好开局。

——经济发展稳健提速。出台扶持民营经济“揭十二条”等政策措施，实施民企双百计划、暖企八大行动，举办揭阳产品全国行（长沙）展销活动、揭阳·深圳工业设计展、第二届玉文化节、揭西第四届生态旅游文化节、普宁第九届衬衣节，推进与俄罗斯、德国、法国等国的经贸合作交流，开展项目建设巡礼周等活动，确保经济平稳较快发展。2012年，全市生产总值1380.15亿元，比上年（下同）增长11.3%；规模以上工业增加值660.4亿元，增长22%；固定资产投资663.51亿元，增长23.9%；社会消费品零售总额521.05亿元，增长15.7%；外贸出口38.1亿美元，增长0.5%；地方公共财政预算收入56.69亿元，增长22.3%。各项存款余额和贷款余额分别增长15.96%、22.48%，存贷比提高至47.2%，均居粤东第一。

——发展引擎日渐形成。狠抓两港经济和重大平台建设，设立空港经济区管委会，完成空港经济区、大南海石化工业园规划，引进和开工一批高端项目，启动空港、海港两大经济引擎。高新区科技企业孵化中心建成使用。“广东省商务生态旅游示范区”功能定位日渐明晰，枢纽产业效应显现。谋划建设中国南方（揭阳）金属生态城，打造英歌山工业园、大北山生态旅游产业园、华侨文化产业创意园等发展载体。项目建设取得重大突破，中委广东石化炼油项目正式开工，惠来电厂3、4号机组核准投产，市重点项目完成投资181.31亿元。

——民营经济逆势转型。实施民营经济转型升级“6166”行动计划，加速传统产业聚合发展。与中科院、北京大学光华管理学院、广东外语外贸大学联合举办榕江培训班，新建、重组行业商协会8个。与中国石油大学等机构开展16项战略合作。获省支持开展民生金融服务试验。启动人才公寓计划，建立市长与高端人才交流日制度，设立榕江人才发展专项基金。普宁服装网络商城成为粤东首个省级电子商务平台。康美药业发布首个国家级中药材价格指数。金属产业创立全国首支由行业协会发起、自筹自建的产业发展基金。

——农业工作扎实拓展。实施农业“一镇一品”工程，创建示范乡镇10个，成立农业产业协会9个、农民专业合作社27个，打造农业品牌14个，在广州举办名优特农产品展销会。引入首农集团设立南方公司。整治农田5.02万亩，开发补充耕地1.94万亩；水利项目投入5.18亿元，恢复和改善灌溉2110公顷，动工建设惠来中东部供水工程。2012年农林牧渔业增加值141.25亿元，增长5.1%；农村居民人均纯收入8046元，增长15.1%。

——城乡建设成效显著。揭东获准撤县设区，市区面积由181平方公里扩大至1031平方公里。榕江北河湿地公园首期工程建成使用，新增公共绿地26.5万平方米、市政道路13.46公里。成功创建省园林城市。新建通村公路317公里，改造国省道157.8公里，港口货物吞吐量1601万吨，机场接送旅客210万人次，“汕潮揭”车辆年票互通互认。“人树一体”理念广为传播，“绿色揭阳”、“生态创富”、“环保为民”工程大力推进，完成碳汇造林26.71万亩、道路绿化1489.75公里，建设生态景观林带126公里、绿色单元600个，节能减排和环境综合整治取得新成效。完成扶贫开发“双到”任务，解决31.77万农村群众的饮水安全问题、5119户农村危房户和520户渔民安居户的住房困难。开展“绿色村庄”、名镇名村创建活动，提高新农村建设水平。

——民生事业小步快跑。全市财政基本公共服务支出71.27亿元，占总支出47.83%；拨付省十件民生实事资金33.97亿元，完成预算317.86%，进度居全省第一。落实市十件民生实事，人大代表、政协委员建议提案办理满意率均达到100%。360多名百岁老人享受政府生活津贴，2000名贫困大学生获得每月200元生活资助。建设公共租赁住房2463套。启动教育创强工作，完成166所规范化学校、173所幼儿园建设。市人民医院创建“三甲”医院，城乡基本医疗保险覆盖率达95%，农村药品供应网和监督网实现全覆盖。“乡乡乐起来”群众文化活动、全民健身运动扎实开展，市广电中心、图书馆建成投入使用，阳美玉都被命名为国家文化产业示范基地。新时期揭阳精神“崇文、尚信、勤行、致远”深入人心。人口计生服务管理水平不断提高。

——社会建设成果丰硕。“三打两建”工作走在全省前列，普宁国际服装城被评为“全国诚信示范市场”，探索康美中药材市场星级药店经营模式，市场经济秩序进一步规范。启动平安揭阳建设，组建公安辅警队伍，刑事、治安有效警情下降10.3%，被省列为首批法治城市，综治信访维稳工作有效加强，安全生产形势稳定好转。成立市社会组织总会等6个服务机构，培育发展社会组织192个，扶持百家示范社团，被省确定为粤东唯一的社会创新实验基地。

——政务环境持续改善。取消、转移、下放行政审批事项154项，公布首批政府购买服务目录。推进电子政务和网上审批，建设市、县行政服务中心。政务参考信息每日常态发送。创新督查方式方法，推动政府百项重点工作落到实处。推进网络问政。加强行政监察和审计监督，设立民营经济服务机构，建立市长特派员制度，大力治庸、治懒、治奢、治散、治浮，全面开展党政机关、事业单位“吃空饷”专项整治行动，以良好的政风促行风带民风。

此外，统计、气象、地震、供销、海洋渔业、妇女儿童、残疾人、双拥、人民防空、人民武装、国防动员、民族宗教、外事侨务、对台事务、新闻出版、广播电视、文史档案、红十字等工作也取得了新进步。

各位代表，过去一年，我们经受了种种困难考验，成绩来之不易。这是省委、省政府和市委正确领导、人大政协监督支持、海内外乡亲关心帮助、全市人民共同努力的结果。在此，我代表市人民政府，向全市广大干部群众致以崇高的敬意！向关心支持揭阳改革发展的社会各界朋友表示衷心的感谢！

在看到成绩的同时，我们也清醒地认识到，当前存在的困难和问题：一是经济弱、财政穷的状况仍未改变，运转提速压力较大；二是发展质量不高，经济转型压力较大；三是资源环境约束趋紧，节能减排压力较大；四是民生事业欠账较多，社会管理压力较大；五是机关效能仍有待进一步提高，等等。对此，我们将高度重视，在加快发展中逐步解决。

2013年工作安排

2013年是全面贯彻落实党的十八大精神的开局之年，也是我市项目建设年、转型升级年、民生改善年、作风转变年。今年政府工作总的要求是：以党的十八大精神为统领，深入贯彻落实省委十一届二次全会和市委五届四次全会精神，紧紧围绕“打造粤东发展极，建设幸福新揭阳”核心任务，以做大经济总量、提高经济质量、建设粤东经济强市和区域中心城市为目标，坚持实干兴市、产业强市，大力实施“创新驱动、项目带动、文化推动、高端拉动、生态联动”发展战略，着力发展两港经济，加快转型升级，走民生引领型发展路子，确保经济平稳较快发展、社会和谐稳定，为全面建成小康社会奠定坚实基础。

今年主要预期目标是：生产总值增长14%以上；规模以上工业增加值增长25%；固定资产投资增长26%；社会消费品零售总额增长18%；外贸出口增长15%；实际利用外资增长15%；地方公共财政预算收入增长16%以上；居民消费价格涨幅控制在3%左右；城镇登记失业率控制在3.5%以内；人口自然增长率控制在7.5‰以内；城镇居民人均可支配收入增长12%，农村居民人均纯收入增长15%。重点抓好八方面工作：

（一）加大产业体系建设力度

坚持“好字当头、能快则快、以快促好”原则，在加快发展中调结构、造引擎、建平台，培育壮大优势产业，着力构建具有揭阳特色的现代产业体系。

推动两港建设提速。海港、空港经济区是揭阳乃至粤东的重大引擎，必须科学规划、加快建设。海港经济区要以石化全产业链及30万吨级产业大港为建设目标，完善规划，科学开发利用土地、岸线、港池资源，以优势资源引进优势项目，以项目集群形成集研发设计、物流、品牌、生产制造为一体的全链条石化产业；同时，以港口为动力源，培育临港制造业和其他配套产业，形成石油化工、电力能源、装备制造和休闲旅游等产业板块。全面启动空港新城建设，加强与驻港八大航空公司深度合作，组织培育民间航空业态，推动空港运输业与区域主要产业的衔接互动及新兴产业的引进，打造以航空物流为先导，以临空型先进制造业和现代服务业为重点的空港产业体系。

加快产业聚合发展。深入实施“6166”行动计划、民企双百计划、暖企八大行动，启动传统产业五年翻番工程，发展战略性新兴产业，构建特色鲜明、充满活力的产业体系。强化行业商协会的功能作为，继续办好企业领军人物培训班，创建产业发展基金，建立产业集聚平台，以产业力量共同抢占“微笑曲线”双高端，形成产业集群转型升级新动力。依托省部支持，对接德国技术，加快中国南方（揭阳）金属生态城规划建设，举办中德工业化城镇化高峰论坛，探索形成传统产业转型升级新模式、园区开发管理新体制、新型工业化与城镇化融合发展新路子，并逐步复制推广至服装、模具、塑料及化工新材料等产业。提高揭阳高新区办园水平，打造战略性发展平台。批量扶持培育产值超十亿、超百亿元龙头企业。力争五年内形成石化、金属、服装、医药、玉器及战略性新兴产业6个千亿产业集群。

优化提升要素配置。推进要素市场建设，突破科技、金融、人才、土地等瓶颈制约，推动产业跨越拓展。与中科院、省科技厅共建科技创新试验区，创立南方科技成果交易平台，建立中德金属科技研究院、中德金属工艺设计院，举办首届科技成果交易暨科技产业招商会。规范提升民营金融业，发展产业金融，力争存贷比年提高3个百分点，建设民生金融试验区。实施企业上市孵化工程，加大力度培育上市公司。强化人才引领发展理念，育才、招才、依托外部人才多措并举，打造人才洼地；加强院会、校企合作，发展职业教育，壮大高技能人才队伍。坚持“以亩产论英雄”，盘活存量土地，优化土地资源配置，保障产业集群用地。

（二）推动经济持续健康发展

全面落实“揭十二条”等政策措施，凝心抓投资，聚力拓市场，扎实促外贸，形成内外需强力拉动经济增长新格局。

强化项目投资拉动。培育大项目，带动大发展。突出抓好去年空港、海港高端招商会和巡礼周等确定的重点项目开工建设。以世界500强、国内行业百强为目标，力争引进一批投资规模超百亿元的项目，推动经济提速增效。

拓展产品消费市场。推进质量强市建设，加强品牌营销，走内涵发展之路。大力发展工业设计。扩大电子商务覆盖面。深化“广东商务生态旅游示范区”建设。抓好“万村千乡”市场工程，办好第三届玉文化节、首届全球金属家俱创意设计大赛等节会，继续推进“揭阳名优产品全国行”，拓展揭货销售渠道。

提高对外开放水平。以行业商协会为主体，建设潮汕“走出去”平台。组织玉器产业协会赴缅甸、外经贸企业协会赴中东、服装纺织协会赴法国、金属企业联合会赴德国开展经贸洽谈活动，组织轻工企业赴俄罗斯开展产品展销活动，鼓励企业到境外拓展市场、投资、收购国际品牌。抓好海港一类口岸基础设施建设，争取设立航空一类口岸、获准对台直航，不断提高开放型经济水平。

加强经济运行监管。保持物价总水平基本稳定，加强粮油等重点商品储备管理。强化统计“四大工程”建设，认真抓好第三次经济普查工作。强化财税征管，实现涉税信息交换共享；严格支出管理，提高财政资金使用效益。深化“三打两建”工作，加快打造法治化国际化营商环境。

（三）突出抓好“三农”工作

落实强农惠农富农政策，加快农业现代化进程，加强社会主义新农村建设，促进农业增效、农民增收、农村发展。

加强现代农业建设。深化“一镇一品”工程，扩大农科院所、拳头产品、龙头企业、协会组织、示范基地、特色品牌、营销体系“七个一”的覆盖面，重点打造20个示范乡镇、20家龙头企业、20个示范基地。组建农业产业联盟，加快首农南方基地建设，推动我市农产品“走出去”。鼓励和引导城市工商资本到农村发展适合企业化经营的种养业，加速传统农业改造和现代农业建设。

加强农村基础建设。发展民生水利，抓好中低产田改造，完善渔业指挥平台建设，改善农村生产条件。改造旧村庄，整治村容村貌，重点推进农村道路、电网、饮水安全工程和危房改造，抓好农村生活垃圾集中处理，建设宜居村庄。谋划新一轮扶贫开发工作，加快脱贫奔康步伐。

增强农村发展活力。推进山区县农村综合改革，加强农村集体资金、集体资产、集体资源“三资”管理，发展壮大农村集体经济。深化农村产权制度改革，开展农村土地确权登记颁证工作，促进农村土地承包经营权流转，提高农民土地收益。扶持发展专业大户、家庭农场、农民合作社等新型生产经营服务主体。

（四）实施城市扩容提质工程

以行政区划调整为契机，实施“市区带动、县城优化、城镇辐射”三大工程，拉开城市框架，突出城市特色，提升城市创富功能、基础设施、人文素质、生态文明，打造宜业宜居城镇群。

提升城市创富功能。依托资源禀赋和产业基础，培育壮大特色产业，探索以产兴城、以城促产、产城一体的产业新城建设新模式，促进新型工业化、信息化、城镇化、农业现代化协调发展，建设区域中心城市。榕城区要着力提升传统产业，大力发展城市服务业，建设岭南特色水城；揭东区要全力创建新型工业化示范基地，建设宜业宜居新城；蓝城区要做大做强玉产业，带动发展文化、旅游等产业；普宁市要加快纺织服装、医药产业集聚发展，打造商贸流通核心区；揭西县要着力发展生态旅游业、生态农业；惠来县要全力服务中委炼油项目建设，规划开发重化工业基地和产业大港；普侨区、大南山侨区要分别对接普宁市和惠来县主导产业开展服务配套，打造华侨文化生态产业区。

提升城市基础设施。改革城市投融资体制，加大市政基础设施投入，完善城市配套功能，提高综合承载和营运能力。加快“三旧”改造步伐，抓好市区30亿、水利市政BT等重点工程，推进碧桂园、恒大集团、正大集团等重点合作项目，改善城市人居环境，提高城市发展品质。完成厦深铁路揭阳段建设任务，实现交通公路投资80亿元，开发利用榕江黄金水道，构建通畅便捷的区域交通网络。规划建设一批特色城镇群，引导农民有序进城，就近就地转移就业，加快城乡发展一体化。扎实推进“汕潮揭”同城化。

提升城市人文素质。弘扬新时期揭阳精神，开展省文明城市、国家卫生城市等创建活动，推进科教、文体、法律、卫生“四进社区”，活跃市民精神文化生活。加强职业道德和社会公德教育，开展“我为城市添光彩”、“争当文明市民”等主题实践活动，提高市民文明素质。加强城市环境综合整治，理顺城市管理和行政执法体制，提高城市管理水平。

提升城市生态文明。推动“人树一体”理念深入人心，继续实施“绿色揭阳”工程，加快国家园林城市和林业生态市创建工作，打造绿道网、生态景观林带等绿色景观，建设黄岐山、紫峰山、桑浦山三大市区生态亮点，广泛植树造绿，让人民群众过上“大树下的幸福生活”，建设健康、美丽揭阳。落实最严格的耕地、“蓝色国土”保护制度和水资源管理制度。加强榕江、练江、枫江污染整治。强化节能减排，完成国家和省确定的污水处理项目建设任务。

（五）大力发展各项社会事业

统筹推进各项社会事业，加快形成政府主导、覆盖城乡、可持续的基本公共服务体系，使发展成果更多更公平地惠及广大人民群众。

全力推进教育创强。优先发展教育事业，完成学前教育三年行动计划，力争义务教育规范化学校覆盖率达到49.7%，高中毛入学率达到88.5%，新增教育强镇23个。建设市直及普宁、揭东、惠来、揭西等5所特殊教育学校。办好揭阳职业技术学院。加强师资队伍培训，提高师德师风建设水平。

大力发展卫生事业。抓好县级公立医院改革试点。加强基层医疗卫生机构标准化建设和一体化管理，巩固综合改革成果。推进全科医生制度建设。公办二级医疗卫生机构配备使用基本药物达到25%以上。实施“阳光用药”制度。继续开展骨干医生“下基层、带徒弟、强医德”活动。

有效提升文体事业。推进文艺精品创作，弘扬潮剧等传统艺术。完善县、镇、村公共文化设施，推进农村电影放映、农家书屋建设、“乡乡乐起来”等文化惠民工程，丰富基层文化生活。抓好市博物馆、市群艺馆、揭阳剧院、观音阁等市级重点文化项目建设。打造阳美玉文化创意产业园、南方（揭阳）潮汕文化产业园等国家、省级文化产业名片。完善体育设施，深入开展全民健身活动。

加强就业和社会保障。制订城乡居民收入倍增计划。加大职业技能培训力度，抓好重点群体就业服务。扩大劳动合同覆盖面，维护劳动者合法权益。开展区域医保创新探索，推进医保异地就医即时结算，实现全市医疗保险基金统收、统管和统支。加强孤残儿童社会保障。

促进各项事业全面发展。强化人口计生服务管理，实施免费孕前优生健康惠民工程。推进气象现代化试点市工作。完成市档案馆新馆建设和《揭阳市志》出版发行。做好国防动员、人民防空、双拥、民族宗教、外事侨务、对台事务、社科、地震、红十字等工作。

（六）深化平安揭阳建设

坚持源头治理、预防在先、法治保障、文化引领，深入推进平安揭阳建设，提高社会管理科学化水平。

维护公共安全。完善社会治安视频监控体系，开展打击“两抢一盗”、“黄赌毒”和整治交通秩序三项行动，大力推进“打控防”一体化建设，着力解决重点地区治安突出问题。严格落实安全生产“一岗双责”责任制，建设突发事件预警信息发布中心，加强食品药品、产品质量等安全管理，及时消除各类安全隐患。

化解社会矛盾。加大矛盾纠纷排查调处力度，加强特殊人群管理，及时化解不稳定因素。落实领导接访和包案制度，高度重视网络舆情，畅通群众表达诉求渠道，做好综治信访维稳工作。

创新社会管理。培育扶持各类社会组织，拓宽参与社会治理空间，充分发挥协同管理作用，建立政府与其他主体多元共治的社会善治模式。做好向社会组织转移政府职能工作，落实政府购买公共服务改革事项。

（七）办好为民惠民实事

把人民幸福作为政府工作的第一追求，集中力量解决群众反映强烈的突出民生问题，努力增进民生福祉。

实施十项民心工程。继续推进揭阳引韩供水、惠来中东部供水、普宁白坑湖水库综合治理三大民生水利工程。抓好汕头至湛江、潮州至惠州、潮州至揭阳、揭阳至惠来等四条高速公路揭阳段建设，推进广梅汕铁路改线工程。建设金属生态城电镀工业园和普宁漂染工业园。

办好30件民生实事。一是城镇低保补差人均月提高60元，农村人均月提高15元，按要求提高五保户供养标准；二是新建公共租赁住房1800套；三是解决4735户农村低收入家庭住房困难；四是完成人才公寓首期百套配置任务；五是治安监控视频采集点增至3万个；六是首期投放公共自行车3000辆，新增公交车100辆；七是完成通村公路硬底化改造300公里；八是完成国道324线普宁池尾至惠来葵潭段、省道335线揭东霖磐立交至揭阳高新区段改建工程；九是完成市区湿地公园二期工程；十是改扩建市区云山殡仪馆；十一是完成18.8万亩高标准基本农田建设任务；十二是实现城乡居民养老保险全覆盖；十三是筹建揭阳市创业带动就业孵化基地，新增城镇就业3.4万人；十四是筹集第二期大学生发展基金1000万元；十五是新建幼儿园49所，改建、扩建86所，新增义务教育规范化学校260所；十六是公办基层医疗机构全面实行平价药包；十七是筹建揭阳高等医专；十八是加快建设市人民医院门急诊住院综合大楼，创建潮汕骨科医院和潮汕妇女儿童医院；十九是建成乡镇文化站12个，村（社区）文化室486个，所有基层公共文化设施免费开放；二十是启动“欢乐进万家”社区文化工程；二十一是选址建设市体校和体育中心；二十二是推广乡镇（街道）农民体育健身工程；二十三是配置食品安全快检车，餐饮单位量化分级管理达到95%以上；二十四是实施价格惠民工程，扩大平价商店覆盖面；二十五是建设生态景观林带169公里，创建绿色单元420个；二十六是完成小型农田水利1个重点县和5个示范镇建设任务；二十七是开展榕江综合治理，启动北河疏浚工程；二十八是筹建粤东残疾人集中就业培训基地；二十九是建设87个乡镇（街道）人力资源和社会保障服务所；三十是每个乡镇（街道）建设一支敬老爱幼志愿服务队。

（八）建设为民务实清廉政府

为民是为政之本，务实是为政之要，清廉是为政之德。我们将始终牢记执政为民的宗旨，践行求真务实的作风，保持清正廉洁的操守，勇于担当，勤勉履职，干净干事，以实实在在的成效取信于民。

深化行政改革。推进市、县大部门体制改革，巩固提高行政审批改革成果。加大督查督办工作力度，发挥市长特派员作用。建成市、县、镇、村四级行政服务体系，打造全省审批项目最少、环节最简、效率最高、费用最低、服务最优的行政办事机构，建设服务型、法治型政府。

践行群众路线。严格执行中央改进作风“八项规定”和省、市实施意见。加大治庸、治懒、治奢、治散、治浮力度，推动行政干部深入基层倾听群众呼声，解决实际问题，严肃纠正损害群众利益等不正之风。自觉接受人大、政协和社会各界的监督，广泛听取各民主党派、工商联、无党派人士和各人民团体的意见建议，促进科学民主依法决策。

打造学习型机关。健全和落实常态化学习制度，形成爱学好学的良好氛围，使学习真正内化为每个干部的一种生活态度和精神追求，成为各级政府不断了解新情况、把握新形势、解决新问题的有力抓手。

加强廉政建设。全面落实党风廉政建设责任制，从严治理领导干部和国家工作人员收送“红包”礼金问题，开展廉政风险防控，强化行政监察和审计监督，注重从源头上防治腐败。厉行勤俭节约，反对铺张浪费。加强廉政文化建设，严格执行廉洁从政各项规定，营造风清气正的良好施政氛围。

各位代表！重任在肩，时不我待。让我们紧密团结在以习近平同志为总书记的党中央周围，在省委、省政府和市委的坚强领导下，高举旗帜，解放思想，改革创新，开拓进取，为“打造粤东发展极，建设幸福新揭阳”、早日全面建成小康社会而努力奋斗！
